# Implementing an early childhood school-based mental health promotion intervention in low-resource Ugandan schools: study protocol for a cluster randomized controlled trial

**DOI:** 10.1186/1745-6215-15-471

**Published:** 2014-12-01

**Authors:** Keng-Yen Huang, Janet Nakigudde, Esther Calzada, Michael J Boivin, Gbenga Ogedegbe, Laurie Miller Brotman

**Affiliations:** Department of Population Health, New York University Langone Medical Center, 227 East 30th Street, 1st Floor, New York, NY 10016 USA; College of Health Science, Makerere University, P.O. Box 7072, Kampala, Uganda; School of Social Work, The University of Texas at Austin, 1925 San Jacinto Blvd, D3500, Austin, TX 78712 USA; Department of Psychiatry and Department of Neurology and Ophthalmology, Michigan State University, 965 Fee Road, Room A227, East Lansing, Michigan 48824 USA

**Keywords:** School program, Mental health, Prevention, Implementation, Uganda, Sub-saharan Africa, Low-income country, Early childhood, Task shifting, Consolidated framework for implementation research

## Abstract

**Background:**

Children in Sub-Saharan Africa (SSA) are burdened by significant unmet mental health needs, but this region has limited access to mental health workers and resources to address these needs. Despite the successes of numerous school-based interventions for promoting child mental health, most evidence-based interventions are not available in SSA. This study will investigate the transportability of an evidence-based program from a developed country (United States) to a SSA country (Uganda). The approach includes task-shifting to early childhood teachers and consists of professional development (five days) to introduce strategies for effective behavior management and positive teacher-student interactions, and group-based consultation (14 sessions) to support adoption of effective practices and tailoring to meet the needs of individual students.

**Methods/Design:**

The design of this study is guided by two implementation frameworks, the Consolidated Framework for Implementation Research and the Teacher Training Implementation Model, that consider multidimensional aspects of intervention fidelity and contextual predictors that may influence implementation and teacher outcomes. Using a cluster randomized design, 10 schools in Uganda will be randomized to either the intervention group (five schools) or the waitlist control group (five schools). A total of 80 to 100 early childhood teachers will be enrolled in the study. Teacher utilization of evidence-based strategies and practices will be assessed at baseline, immediate post-intervention (six months after baseline), and at seven months post-intervention (during a new academic year). Fidelity measures will be assessed throughout the program implementation period (during professional development and consultation sessions). Individual teacher and contextual factors will be assessed at baseline. Data will be collected from multiple sources. Linear mixed-effect modeling, adjusting for school nesting, will be applied to address study questions.

**Discussion:**

The study will produce important information regarding the value of an evidence-based early intervention, and a theory-guided implementation process and tools designed for use in implementing early childhood evidence-based programs in SSA countries or resource-constrained community settings.

**Trial registration:**

This trial was registered with ClinicalTrials.gov (registration number: NCT097115) on 15 May 2013.

## Background

Children in Uganda make up about half (56%) of the total population (compared to 20% in the United States) [1], and face many physical, mental health and educational challenges [1, 2]. Most Ugandan children live in disadvantaged communities with high rates of chronic poverty (38%), domestic violence (30%), physical violence toward children (80%), depression (33 to 39%), malaria (70 to 80%) and HIV or AIDS (6%) [3–9]. These issues have led to a significant number of orphans, poor child mental health and numerous child welfare issues [1, 8]. Poor child mental health has been shown to be strongly related to poor physical health, low secondary education enrollment, substance abuse and violence among adults in developing countries [10–12], and has enormous consequences on the country’s economic development and international resources.

Although Uganda receives a large amount of international resources to address many child health needs, most focus on the provision of clean water, vaccinations, nutrition programs, protection and education [13]; limited attention has been given to child mental health problems. Of the available mental health interventions, most are limited to awareness or public education campaigns [14], and no attention has been paid to developing a population-based strategy to promote child mental health. This service gap is partly attributable to a lack of trained child mental health workers. Uganda, like many other developing countries, has few mental health workers (0.08 psychiatrists, 0.01 psychologists, 0.01 social workers and 0.78 nurses per 100,000 population, compared to between 10 and 60 in developed countries) [15]. Most mental health professionals are not specialized or trained in prevention services, and Uganda’s mental health policy has remained at the drafting stage since 2000 [16].

### Population-level approach to promoting child mental health

A large body of developmental research suggests that preventive interventions during early childhood can result in improved mental and physical health, academic attainment and overall wellbeing in children [17, 18]. Although numerous evidence-based preventive interventions for young children have been implemented in developed countries [17, 19–21], such mental health programs are usually not available in Uganda or Sub-Saharan Africa (SSA) [22]. Disseminating evidence-based programs (EBPs) to SSA has the potential to be a cost-efficient approach to addressing the enormous mental health needs of the pediatric population.

To have a larger public health impact and to maximize reach to the pediatric population, one strategy is to provide interventions in the school setting [23]. In Uganda, 84% of children are enrolled in primary school, with most (70%) entering at five or six years of age (Primary one) [24]. Universal intervention (for all children) in the school setting provides an opportunity to serve all students and has been shown to be an effective approach in addressing health problems (such as HIV and AIDS and poor nutrition) in SSA countries [25, 26], as well as improving child mental health in developed countries [27–30].

Given the limited number of mental health professionals in Uganda (and most SSA countries), it is not feasible to rely on such professionals to implement mental health preventive interventions (as is often the case in developed countries). Task-shifting, endorsed by the World Health Organization, offers a solution to overcoming this human resource barrier. Task-shifting involves redistributing tasks from professionally trained workers to those with less training and fewer qualifications [31, 32]. Under the right conditions (for example, political support, embedded into the community, appropriate training and coaching, remuneration and incentive systems), this approach can lead to significant health gains [33, 34]. For developing a school-based program in Ugandan schools, task-shifting involves redistributing responsibilities for implementation from mental health workers (such as social workers or psychologists) to teachers. Successful task-shifting will involve: 1) modifying the intervention to be delivered by teachers, 2) training the teachers in the mental health preventive intervention and 3) providing adequate consultation and support so that the teachers implement the intervention with fidelity. Adapting efficacious programs from developed countries to SSA countries using task-shifting also requires thoughtful consideration of individual- and system-level factors (such as leadership support, school climate and teacher characteristics) that may influence effectiveness of task-shifting or program implementation outcomes [33, 35–38]. Although some school health interventions conducted in high-income countries have suggested that leadership support, school climate and teacher characteristics may impact intervention effects [33, 35–38], we are not aware of any other studies that examine these implementation questions in African schools. The systematic study of factors that may influence implementation is necessary to develop implementation strategies to ultimately impact population health in African children.

The current protocol seeks to test a potentially cost-effective population approach to address child mental health needs in Uganda by adopting an EBP that utilizes universal prevention, task-shifting and school-focused approaches from a developed country to a SSA country (Uganda). This protocol focuses on the evaluation of the process and impact of such an implementation approach. In addition, this study will systematically examine individual and system level factors that may enhance or hinder program implementation quality and outcomes. This information is critical to the development of additional strategies to enhance task-shifting in program implementation and program outcomes.

### The mental health intervention

The EBP that we apply in this study is ParentCorps. ParentCorps is a culturally-informed, multicomponent intervention for young children that promotes high-quality learning environments at home and school (such as positive parent-child and teacher-child interactions and effective behavioral management practices), resulting in meaningful educational, developmental and health benefits for children, especially those who are behaviorally dysregulated (for example impulsive or inattentive) in early childhood [39–42]. ParentCorps was built on an extensive body of cross-cultural parenting and child developmental research [11, 39, 42–47]. The program includes three core intervention components. The ‘Professional Development’ component aims to increase knowledge, motivation and capacity to employ evidence-based practices for creating positive teacher-child interactions and strong home-school connections, and for implementing the programs for parents and students. Professional Development includes the ParentCorps ‘FUNdamentals’ course (four days large-group experiential training and two additional days of separate training for mental health professionals in implementing the ‘Program for Parents’ and teachers in implementing ‘Program for Students’) and ‘Coaching’ (providing *in vivo* support to trainees to enhance their ability to identify and address the needs of students and to employ evidence-based practices in daily interactions with students and parents). The ‘Program for Parents’ component (consisting of 14 two-hour sessions) aims to increase parent knowledge, motivation and capacity to use a set of evidence-based parenting practices (parallel to those presented in Professional Development). The ‘Program for Students’ component (consisting of 14 two-hour sessions) aims to increase children’s knowledge, motivation and capacity to use social-emotional and behavior regulation skills through lessons, activities and a systematic approach to behavior management. In two cluster (school) randomized controlled trials (RCTs) with low-income, minority students in New York City in the United States, ParentCorps has shown to be highly acceptable to ethnically diverse populations in poor urban communities, and has had significant, robust and sustained effects on all targeted teacher, parent and child outcomes [39–41, 48–50]. These include effects on teachers’ utilization of proactive and positive reinforcement strategies, positive classroom climate and confidence in engaging families (Cohen’s ds ranged from 0.42 to 0.85, comparable to the range 0.51 to 1.29 reported in the literature [51]); parents’ utilization of effective parenting practices, involvement in child education and parenting knowledge (Cohen’s ds ranged from 0.16 to 0.50, compared to 0.30 to 0.88 reported in the literature [52]); and early childhood students’ social-emotional (adaptive behaviors, externalizing and internalizing problems) and academic outcomes (Cohen’s ds ranged from 0.25 to 0.56, compared to 0.24 to 0.81 reported in the literature [52–55]).

In the United States, mental health professionals have been responsible for implementation of the Program for Parents, while teachers are responsible for the Program for Students. Using a task-shifting strategy to bring ParentCorps to Ugandan schools, the long-term plan is for Ugandan teachers to implement the Program for Parents as well as the Program for Students. A systematic series of studies with Ugandan teachers, families, school leaders, mental health professionals and policymakers and key stakeholders across Uganda indicate that the ParentCorps implementation model (Programs for Parents, Programs for Students, and Professional Development) were a good fit, and the teachers are motivated to implement both intervention components. For example, previous studies found that: 1) the task-shifting model is a familiar concept, as most schools (90%) and teachers (72%) have experience in child health promotion activities; 2) Ugandan teachers report similar mental health risk factors as those identified in other countries, including lack of parental supervision and/or involvement, negative parenting, poverty, coming from a broken home and disadvantaged home and neighborhood environments (such as slum areas and exposure to violence). Teachers also express that effective preventive strategies should include effective discipline, positive teacher-child and parent-child relationships, education of parents and children and promotion of parental involvement; 3) most teachers are interested in ParentCorps training (99%), and in providing mental health promotion activities to students and parents (97%) (K-Y Huang and J Nakigudde unpublished observations).

As the first step of transporting ParentCorps to Uganda, this initial study will focus on the teacher Professional Development component only. As in the United States, mental health professionals will provide Professional Development (ParentCorps FUNdamentals and Coaching) to implementers (in this case, teachers). This study will evaluate the feasibility of training, coaching and supporting Ugandan mental health professionals from a distance to provide Professional Development for Ugandan teachers, and estimate the impact on teacher practices.

### Conceptual framework for implementation

**Figure 1:**
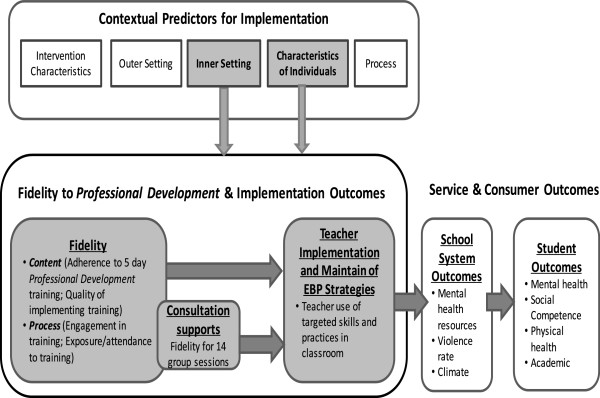
**Implementation conceptual model.** This Figure illustrates the relationship among contextual predictors, implementation fidelity and implementation and child mental health outcomes.

Figure 1 shows the theory of change model for ParentCorps implementation. Our implementation framework is adapted from Reinke *et al*.’s Teacher Training Implementation Model (TTIM) [56] and Damschroder and Lowery’s Consolidated Framework for Implementation Research (CFIR) [36]. While TTIM focuses on multidimensional aspects of intervention fidelity as related to implementation outcomes, CFIR focuses on five domains of contextual factors that may impact implementation outcomes. As per the model, fidelity (including adherence and quality of program implementation, and trainees’ engagement and exposure to the program as defined in the TTIM [56]) may impact teacher implementation of evidence-based practices in the classroom, which in turn will impact student and school outcomes. In addition, five domains of contextual predictors, derived from the CFIR [36] (including intervention characteristics, outer setting, inner setting, individual characteristics and organization process), may impact program implementation (both on fidelity and implementation outcomes) and sustainability. This study focuses on implementation processes and outcomes in the gray shaded boxes in Figure 1. Among the contextual predictors, only the school/inner characteristics (such as school leadership and climate) and individual characteristics (such as teacher factors) will be considered because of the relevance to the school contexts. This study will not focus on contextual predictors related to intervention (ParentCorps) characteristics’ because previous work shows that ParentCorps fits well within Ugandan contexts (see above).

### Implementation aims and hypotheses

***Firstly, we aim to evaluate the implementation fidelity of Professional Development*(*ParentCorps FUNdamentals and Coaching*) for Ugandan teachers when implemented by Ugandan mental health professionals.** We hypothesize that Ugandan mental health professionals will implement Professional Development with high levels of fidelity.

**Secondly, within the context of an RCT, we aim to evaluate the impact of Professional Development on teacher practices in the classroom during the training school year, and during the following school year with different students.** We hypothesize that, relative to teachers in schools randomized to the waitlist control condition, teachers in intervention schools will use more effective practices in the classroom and these practices will be maintained over time and with a different student population.

**Thirdly, within intervention schools only, we aim to examine the relation between implementation fidelity, context (individual and system factors) and teacher practices.** We hypothesize that higher levels of implementation fidelity will be associated with greater use of effective practices in the classroom, and that positive school and teacher characteristics (such as better school leadership, and higher teacher self-efficacy) will predict higher teacher engagement in Professional Development and greater use of effective practices in the classroom overtime.

## Methods/Design

### Design

This study applies a matched-pair cluster randomized waitlist controlled design. To ensure approximately similar characteristics in the intervention and control conditions, a statistician unfamiliar with the study schools will match the schools based on size (number of teachers and students). Five pairs of schools will be identified. Schools will be selected from a previous study of children’s health (K-Y Huang and J Nakigudde unpublished observations), which randomly selected 30 schools from the schools registered under Ministry of Education in Kampala, Uganda. The 10 schools for the RCT will be selected from this pool of 30 schools because they all indicated interest in participation. Within each pair, one school will be randomly assigned to intervention, and the other to the waitlist control condition (receiving intervention after the RCT post-assessment). By the follow-up assessment in intervention schools, all 10 schools will have received intervention. The study has been approved by Institutional Review Boards (IRB) of New York University Langone Medical Center (IRB number: S13-00362), Makerere University (IRB number: SBS110), and Ugandan National Science and Technology (IRB number: SS3194).

### Participants

The study aims to enroll primary school and all early childhood teachers, including nursery pre-kindergarten and kindergarten, and primary one, two and three (first to third grades), serving students between four and eight-years-old. Based on our prior work, Ugandan primary schools have, on average, about 700 students (ranging from 100 to 2,300, SD = 440), 23 teachers (ranging from 7 to 56, SD = 13) and 54 students in each class (ranging from 15 to 90, SD = 21). The schools have on average six to eight early childhood teachers (for four to eight-year-olds). We anticipate that 80 to 100 early childhood teachers from the 10 study schools (40 to 50 teachers in each condition) will participate in the RCT, and estimate that more than 3,200 children across conditions (including waitlist control schools at the end of the trial) will be impacted during the study period (Huang and Nakigudde, unpublished observations). Although the trial is carried out in schools, parents, guardians and students are not considered subjects of this study. They will not participate in any research activity, therefore, consent of parents and guardians will not be obtained.

The study is being conducted by investigators from the United States and Uganda. All study recruitment and assessment procedures will be conducted by Ugandan research staff. Principals will be informed about the randomization procedures, study timeline, teacher assessments and intervention procedures (including immediate provision in the intervention schools and delayed provision in the control schools). Once principals agree to participate and sign a written agreement, teachers from the schools will be approached by research staff to assess their interest. Teachers will provide written informed consent and will be informed that they have the right to refuse to participate in the study, even though the principals and other teachers agree (with no negative consequences).

A total of 10 Ugandan mental health professionals will be recruited and trained to implement ParentCorps Professional Development. Inclusion criterion for mental health professionals is: psychologists, social workers and mental health counselors educated to at least master’s degree level,, or psychologists, social workers, counselors and psychiatric nurses educated to at least bachelor’s degree level with at least three years of clinical experience. They will be recruited from local Universities or mental health facilities. Mental health professionals who agree to participate will provide written informed consent, which will allow the research team to gather fidelity data (self-reported or observational data) from them.

### Sample size and power

We conducted power analyses for teacher outcomes (the primary evaluation outcomes), assuming intention-to-treat analyses will be applied. Power analysis for aim three (contextual moderators) is not computed given the exploratory nature and lack of empirical evidence in this area of research. In cluster match-pair randomized designs, in addition to the usual study design parameters, the effect sizes detectable with 80% power of two-sided tests with level of significance α = 0.05 depend on the cluster (school) sizes (m), the intra-class correlation (ICC) associated with the cluster and the correlation between the schools within a pair (ρ). In technical terms, the clustering leads to a decrease of the actual sample size (N) by a factor called variance inflation factor (VIF = 1 + (m-1) ICC). The effective sample size (N*) in the case of cluster randomization is N* = N/VIF. Akin to a paired t-test, randomization within matched pairs can increase the power of a test, especially if ρ is large. Depending on the analytic model, the magnitude of the detectable effects can also be varied. For example, a model which includes covariates that account for some variance in the outcome, such as baseline levels of the outcome, would be more powerful (and would provide adequate power to detect smaller effects) than a model that does not make use of covariates [57].

We estimate the magnitude of the effects that are detectable with 10 schools, matched in five pairs, and 80 to 100 teachers (or eight to 10 teachers per school) with respect to post-intervention outcomes, at 80% power of a 2-sided test with level of significance α = 0.05. The following assumptions are made in those computations: 1) the baseline values of the outcome and other baseline covariates in the model account for 20 to 50% of variance of the post-intervention outcomes (estimated variance obtained from trials conducted in the United States); 2) correlations of the outcome measures between schools within a pair (ρ) will be high, ranging from 0.50 to 0.70; and 3) ICCs associated with clustering of teachers within a school will range from 0 to 0.10. The detectable effects (Cohen’s d) would be d = 0.11 to 0.29. The proposed study will have sufficient power to detect impact on teacher outcomes that are meaningful and realistic.

### Description of the intervention

To implement the intervention, a ‘train the trainer’ model was employed. Three Ugandan mental health professionals spent 10 days in the United States at ParentCorps Academy. Upon return to Uganda, they facilitated training of their peers (seven mental health professionals) along with 10 hours of video conference support from ParentCorps Academy. This group of 10 mental health professionals, led by the three original trainees, is responsible for the implementation of ParentCorps FUNdamentals. All trained mental health professionals are responsible for implementing Coaching for teachers. The mental health professionals will participate in 14 group supervision calls with ParentCorps Academy over the course of the implementation phase of the study (or on the week they provide coaching sessions to the teachers).

#### Intervention group

For this study, intervention is limited to Professional Development (described above). Because the plan was to wait to implement the Programs for Parents and Programs for Students until after the completion of this initial study, the two-day training of ParentCorps FUNdamentals that are meant to train mental health professionals and teachers to implement the programs with fidelity will not be included. FUNdamentals has been ‘repackaged’ to be delivered to Ugandan teachers by Ugandan mental health professionals over a five-day period. As in the version implemented in the United States, FUNdamentals is a large-group experiential training series aimed at building knowledge, evidence-based skills, shared language, motivation for change and a sense of community. Over the five days, in a safe and supportive environment, participants are asked to reflect on their assumptions (positive and negative) about parents and students and to connect those assumptions to their current practices and capacity to help children succeed. Under this learning environment, they also learn a series of evidence-based behavioral management skills. Coaching sessions provide support for teachers to employ evidence-based practices in daily interactions with students and parents, and helps teachers to identify and address the needs of students without the requisite self-regulation skills for school success. The Coaching model (one-to-one professional to teacher ratio in the United States) was revised to be delivered by a pair of mental health professionals to groups of early childhood teachers at the school during school breaks or after school hours because of the lower cost and potential benefits from working as a group in a collective culture (gaining support and opportunities to learn from one another). Based on feedback from school leaders, 14 group coaching sessions (one to two hours duration) were designed to take place over a six-month period, decreasing in frequency over time (eight weekly sessions, four bi-weekly sessions and two monthly sessions).

### Study measures

This study utilizes a multi-method and multi-informant approach to measurement. Data will be collected from teachers, mental health professionals and objective blind observation (video tape coding). The measurement strategy was developed under the Reach/Efficacy-Adoption/ Implementation/Maintenance (RE-AIM) evaluation framework [58, 59]. The RE-AIM framework conceptualizes the public health impact of an intervention as a function of five dimensions: reach, effectiveness, adoption, implementation and maintenance. The dimensions that are captured in the RE-AIM are consistent with the constructs described in our implementation conceptual framework.

#### Reach

Early childhood teachers will be characterized in terms of demographics, attitudes about use of evidence-based practices and students, parents and school leadership [60, 61], self-efficacy [62] and motivation for change [63].

#### Effectiveness

Teacher practices (behavioral management practices and classroom climate) will be assessed by self-report [64] and classroom observations. The Teacher Instructional Practices and Processes System, a classroom observation coding system designed to assess climate, instructional practices and processes for use in schools in developing countries (TIPPS) [65], will be applied. Two trained observers, who are masked to the study condition, will conduct live observations of teachers for a 20-minute period during a lesson. Observers will rate each teacher’s behavior independently. Effectiveness data will be collected before intervention and immediately after intervention.

#### Adoption

To characterize the target setting (school inner setting), data will be collected at baseline from two sources. School principals will complete a School Environment Questionnaire [66] that assesses school demographics, resources and school climate. Teachers will complete a School Climate Questionnaire [67] that measures principals’ leadership, cohesion, communication, stress and autonomy.

#### Implementation

Four domains of implementation fidelity will be assessed. Adherence, the extent to which the mental health professionals deliver the core intervention content and as per program guidelines, will be evaluated based on ParentCorps’ integrity checklists completed by the implementer and videotapes and/or audiotapes of FUNdamentals (all five days) and coaching sessions (five of 14) coded by ParentCorps Academy faculty. Quality of program implementation will be assessed based on teacher satisfaction with the Professional Development and ratings of coaches’ competence. Engagement will be evaluated with two ParentCorps measures of knowledge and practice [40]. Level of knowledge improvement from pre- to post-training will be used as indicators for level of engagement. Exposure will be measured by attendance at ParentCorps FUNdamentals (five days) and coaching sessions (14 sessions).

#### Maintenance

Teacher practices during the new school year (about seven months after the intervention) will be measured via self-report and classroom observations as described above.

### Statistical methods

#### Preliminary analyses

Baseline equivalence between intervention and control schools will be examined. Missing data patterns and distribution of the study variables will be inspected prior to any outcome analyses. In addition, psychometric properties of study measures will be inspected, and only measures with adequate reliability and validity will be applied for subsequent study. For measures that evaluate similar constructs, composite scales will be created (to minimize number of analyses).

#### Analyses for aim one

To characterize the quality of implementation we will carry out a series of descriptive analyses and examine level of fidelity in four domains (described above). Implementation in intervention schools and in waitlist control schools (during the implementation phase) will be examined. It is possible that implementation in waitlist control schools will be of higher quality since this will be the second opportunity for implementation after a year of experiences, supervision and support. We will examine the two groups separately first, and then combine if no meaningful differences are found.

#### Analyses for aim two

To evaluate impact on teacher practices, we will apply linear mixed-effect models (apply SAS PROC MIXED procedure [68]) that will adjust for school nesting [69]. We will model post-intervention teacher practices (time two (immediate post intervention or six months after baseline) as a function of intervention, adjusting for time one teacher practices. To evaluate whether teacher practices are maintained over time (time three, seven months after post intervention or the first term of new school year), similar mixed-effect models will be applied. However, only the intervention sample will be analyzed (given that the waitlist control teachers will have received the intervention by time three). We will use repeated data over time (times two and three). We will model teacher practices post-intervention as a linear function of time, adjusting for baseline practices. In addition to random intercepts and slopes for individual teachers, the model will include random effects for schools. If the time effect is not significant, this will indicate maintenance of impact.

#### Analyses for aim three

To study the association between quality of implementation and teacher practices, we will examine whether four domains of fidelity predict change in teacher practices from time one to two. We will use the analytical model similar to the one described above, and model teacher practices at time two as a function of four fidelity measures, controlling for time one teacher practices. To understand how school (inner setting) and teacher (individual characteristics) contextual factors influence teachers’ engagement, exposure and practice, two sets of analyses will be conducted (one for school setting and one for teacher characteristics). Three outcomes (engagement, exposure and change in score on practices) will be analyzed separately. We will model outcomes as a function of school characteristics (such as, leadership, climate and communication) or teacher characteristics (such as self-efficacy).

## Discussion

Children in Uganda and other SSA countries are burdened by significant unmet mental health needs. Knowledge and resource barriers and shortage of mental health professionals limit the capacity of SSA countries to implement preventive strategies or provide basic mental health services. Task-shifting of mental healthcare duties from professionals to teachers is a potentially cost-efficient strategy for mitigating system-level barriers to optimal child mental health in SSA. Although numerous EBPs have been found to meaningfully improve the lives of children living in disadvantaged environments in developing countries, these EBPs have not been transported to SSA, and the implementation supports and adaptations for successful implementation are not well understood. Also, contextual factors that may impact task-shifting or quality of EBP implementation are rarely studied. In order for task-shifting strategies to be effective and translation of EBPs to low-resource countries to be successful, evidence about the quality and process of program implementation and factors predicting task-shifting and implementation outcomes are paramount. This study addresses these implementation and translational research gaps by examining EBP transportability (to developing countries) and effectiveness, and systematically studying factors that may contribute to effective task-shifting. Our implementation study has the potential to illuminate the processes and complexities associated with a task-shifting approach to preventive intervention. Furthermore, this study applies implementation and population health frameworks to guide preventive intervention to address mental health needs in Uganda. If the approach and findings from the proposed study are successful, this study will represent the first step in a series of efforts to transport evidence-based interventions to SSA. Knowledge gained from this study can also be applied to guide other EBP dissemination and implementation efforts that utilize task-shifting strategies in SSA countries.

## Trial status

The study began in July 2013. The first six months was a preparation period for obtaining IRB approval, intervention adaptation, training Ugandan mental health professionals and recruitment of schools and teachers. Data collection in 10 schools and implementation in five schools began in January 2014. Data collection is underway. Participant recruitment is expected to end in December 2014. Data have not yet been cleaned or finalized for evaluation.

## Abbreviations

CFIR: Consolidated Framework for Implementation Research
EBP: Evidence-based program
ICC: Intraclass correlation coefficient
RCT: Randomized controlled trial
RE-AIM: Reach/Efficacy-Adoption/ Implementation/Maintenance
SSA: Sub-Saharan Africa
TTIM: Teacher Training Implementation Model
VIF: Variance inflation factor.
